# Determining Frequency of Multiple Organ System Involvement and Concurrent Lesions Identified in Feedyard Mortalities and Potential Associations with Cattle Demographics

**DOI:** 10.3390/vetsci12070666

**Published:** 2025-07-15

**Authors:** Madeline R. Mancke, Brad J. White, Eduarda M. Bortoluzzi, Brandon E. Depenbusch, Paige H. Schmidt, Rachel E. Champagne, Makenna Jensen, Phillip A. Lancaster, Robert L. Larson

**Affiliations:** 1 Beef Cattle Institute, Kansas State University, Manhattan, KS 66506, USA; 2 Department of Anatomy and Physiology, Kansas State University, Manhattan, KS 66506, USA; 3 Irsik and Doll Feed Services, Inc., Cimarron, KS 67835, USA

**Keywords:** feedyard, cattle, necropsy, lesions, concurrent

## Abstract

Necropsies are commonly used to diagnose the causes of death in feedyard cattle, but the documentation of multiple organ system involvement and concurrent lesions is limited. This observational study aimed to determine the frequency of such findings and their associations with animal demographics. Systemic necropsies were conducted for 889 cattle mortalities with minimal autolysis across six feedyards in the Central High Plains during the summers of 2022 and 2023. Lesions and abnormalities were recorded along with arrival weight, sex, days on feed (DOFs), and number of treatments. The results showed that 72% of mortalities had more than one gross lesion, averaging 2.3 lesions per animal. The most common organ systems affected together were digestive and pulmonary (19%), followed by cardiovascular, digestive, and pulmonary (6%), and cardiovascular and pulmonary (5%). Common concurrent lesions included bronchopneumonia with an interstitial pattern (BIP) and gastrointestinal lesions (GI) (8%), bronchopneumonia and GI (7%), and acute interstitial pneumonia (AIP) and GI (3%). A generalized linear mixed effects model revealed that the likelihood of multiple lesions increased with DOFs (*p* = 0.02). These findings highlight the value of thorough necropsy documentation to enhance our understanding of disease and guide improved feedyard management and treatment practices.

## Simple Summary

This study examined the necropsies of feedyard cattle to determine how often multiple organ systems and concurrent lesions were involved in mortalities. Researchers analyzed 889 necropsies from six feedyards in the summers of 2022 and 2023. They found that 72% of the cattle had more than one lesion, with an average of 2.3 lesions per animal. The most commonly affected systems were the digestive and pulmonary systems. The likelihood of multiple lesions increased with a greater number of days on feed. The findings suggest that recording all abnormalities during necropsies could help improve cattle health management and treatment strategies.

## 1. Introduction

Feedyard mortalities impact animal welfare, profitability, and overall production [1]. Factors that may influence the health of feedyard cattle include but are not limited to the following: arrival weight, distance traveled and stressors associated with transportation, commingling, and extent of the feedyard personnel’s experience in animal care [2,3,4]. Studies in the previous literature focused on beef cattle found the incidence of overall morbidity rates to be 15–45% and overall mortality rates to be 1–5% [1,5,6]. Feedyard personnel often perform field necropsies on mortalities to evaluate and identify causes of death [7]. The necropsy methodology varies among operations ranging from not performing a necropsy, performing a cursory examination, or completing a thorough necropsy. Necropsy evaluations often result in a single final diagnosis for each animal, though necropsies should not be concluded upon finding a severe disease process.

Though mortalities are often attributed to a single disease process, multiple pathologic processes may contribute to diseases of feedyard cattle. Pulmonary disease continues to be the leading cause of feedyard mortality [8,9,10]. Gastrointestinal diseases, such as bloat, are also common causes of death [11]. Cardiovascular disease is being reported with increased frequency in feedyard mortalities, and this could be due to increasing prevalence or increasing evaluations and awareness of the disease [12]. The frequency of singular disease processes has been established, but little information is available describing multiple organ system involvement or concurrent lesions of feedyard mortalities. Understanding the frequencies of concurrent lesions and potential associated risk factors could help identify diseased cattle ante mortem and facilitate the creation of more accurate treatment plans.

Due to limited research on multiple coexisting organ system involvement or concurrent lesions in feedyard cattle, producers are forced to focus on one disease in isolation from other diseases and adjust preventative and therapeutic plans accordingly. The primary study objective was to quantify the frequency of common concurrent pathologic lesions and organ system (pulmonary, gastrointestinal, cardiovascular, and other) involvement in feedyard mortalities. The second study objective was to determine potential associations between the probability of concurrent lesions or effects on organ systems with demographic factors (sex, days on feed, arrival weight, and number of treatments).

## 2. Materials and Methods

All procedures and observations were performed after the cattle’s deaths. The Kansas State University Institutional Animal Care and Use Committee (IACUC) was contacted but deemed a protocol unnecessary due to no procedures being performed on live animals.

This cross sectional, observational study was designed to determine the frequency of multiple organ system involvement and concurrent lesions in feedyard cattle mortalities throughout the feeding phase. In the months of June and July of 2022 and 2023, systemic necropsies were performed on feedyard cattle in six Central High Plains feedyards. Necropsies were performed by trained technicians through Kansas State University’s Beef Cattle Institute. Enrollment into the study included cattle that were necropsied within 18 h of death with minimal to no autolysis. Following necropsy, treatment history and animal demographics were provided by the feedyard staff.

A systemic necropsy was performed on every mortality that qualified for study enrollment. Brain tissue was not examined due to logistical constraints concerning the rendering company disposing of the animals. Individual animal identification was recorded, and a case number was created for each mortality. A standardized necropsy evaluation form was used, in which all findings were recorded in a systematic fashion by recording designated abnormalities from a list for each area examined (Table 1). All necropsies were performed in a similar fashion, and the order of evaluation of organ systems was consistent among cases. To ease the removal of the animal, a necropsy technique was used to minimize the number of detached organs, similar to the previously published literature [13].

An external examination was performed first, recording sex, extent of autolysis, and flesh category (degree of fat cover). The musculoskeletal system, including extremities, was then examined. The tongue was removed, and the oral cavity was examined. The esophagus, trachea, and larynx were opened and examined.

After opening the chest cavity, the heart was completely removed and examined for the presence of pericardial effusion, determination of the heart shape, and observation of the external myocardial surface. Heart shape scores were recorded as 1–5 (1 = normal, 2 = mild changes, 3 = moderate changes, 4 = severe changes, and 5 = severe changes with flaccid heart), following a previously published scoring system [14]. The heart was transected horizontally to examine papillary muscles and endocardium. All valves were examined for potential signs of endocarditis. The lungs were then removed from the thoracic cavity so that all eight lobes were visualized and percent involvement of pathology was estimated. Lung size, texture determined by palpation, and presence of pleural effusion, adhesions, abscesses, lungworms, or any acute or chronic disease processes were recorded.

The gastrointestinal tract was examined, including abomasum, rumen, small and large intestine serosa, mucosa, and content. Rumen scores were recorded as 1 through 5 (1 = normal—no evidence of scar tissue or active lesions; 2 = parakeratosis, abnormal papillae, and presence of clumps of papillae; 3 = one or more healed scars; 4 = mixture of one or more healed scars and active lesions; and 5 = one or more active lesions) based on an original scoring system that was created. In the abdomen, multiple areas were examined including mesenteric lymph nodes, liver, gallbladder, kidney, bladder, reproductive system, and spleen. Observations were made regarding the presence of peritoneal fluid, adhesions within the abdomen, and liver abscesses. Liver abscess scores were recorded as O (no abscesses), A (one or two small abscesses or up to four grouped abscesses that are usually less than an inch in diameter and surrounded by healthy liver), and A+ (one or more large abscesses greater than an inch in diameter, along with inflammation of the liver tissues around the abscesses), as seen in the previously published literature [15].

During the full systemic necropsy, all lesions were recorded, and a final diagnosis was generated based on all findings. Lesions were described as any gross abnormality noted in the necropsy examination. All necropsies were performed by a trained research team, and all lesions and diagnoses were confirmed by a veterinarian through Kansas State University’s Beef Cattle Institute. The final diagnosis was based on lesion severity and organ system affected to determine potential contribution to the fatality. A final diagnosis of multiple causes was assigned when two or more lesions that could have contributed to fatality (e.g., heart disease and acute interstitial pneumonia) were identified.

**Figure 1:**
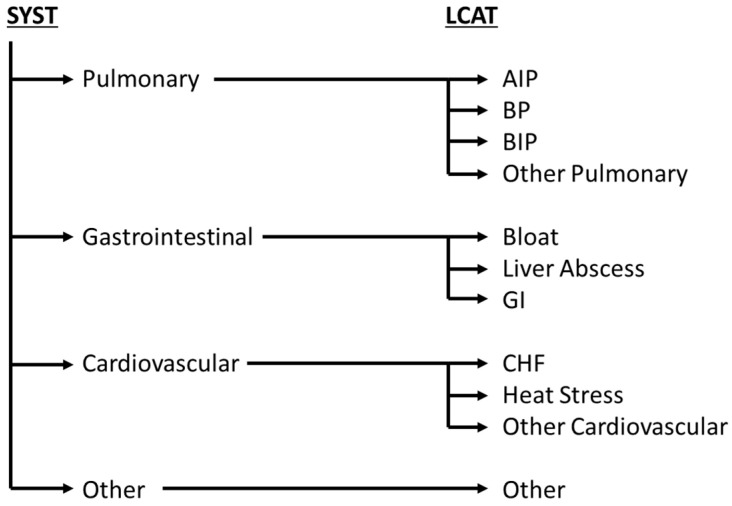
Organ systems affected (SYST) and lesion categories (LCATs) on 889 Central High Plain feedyard mortalities at gross necropsy in the summers of 2022 and 2023. Abbreviations in the figure include AIP (acute interstitial pneumonia), BP (bronchopneumonia), BIP (bronchopneumonia with an interstitial pattern), GI (gastrointestinal), and CHF (congestive heart failure).

Each lesion or abnormality was classified to an organ system (SYST). The SYSTs included the following: pulmonary, gastrointestinal (GI), cardiovascular, and other (Figure 1). Within each SYST, lesions were categorized into lesion categories (LCATs). There were multiple LCATs for each SYST: four for the pulmonary SYST, three for GI SYST, three for cardiovascular SYST, and all miscellaneous LCATs going into other SYST. Pulmonary LCATs included acute interstitial pneumonia (AIP), bronchopneumonia (BP), bronchopneumonia with interstitial pneumonia (BIP), and other pulmonary lesions. The BIP lesions represent two separate disease processes (bronchopneumonia and interstitial pneumonia) [16,17], but their occurrence together was recorded as a single lesion. Other pulmonary lesions included all other respiratory diagnoses: embolic pneumonia, fibrinonecrotic larynx, granulomatous pneumonia, lung abscess, lung blood clot, lung nodules (small mass of dense tissue), pleuritis, and pulmonary hemorrhage (bleeding from the lungs). Gastrointestinal LCATs included bloat, liver abscesses, and other GI lesions (colon ulcers, congested liver, diaphragmatic hernia, frothy rumen, hemoperitoneum, hemorrhagic/red abomasal mucosa, hemorrhagic/red rumen mucosa, intestinal strangulation, jaundiced liver, liver adhesions, liver flukes, liver laceration, liver nodules (small mass of dense tissue), liver scars, ruminal parakeratosis, and peritonitis). Cardiovascular lesions included heat stress, congestive heart failure (heart failure, right ventricular concentric hypertrophy; CHF), and other cardiovascular lesions (anemia, endocarditis, LV concentric hypertrophy, myocarditis, nutmeg liver, and pericarditis). Other lesions included dystocia/calving injury, jaundice, kidney stones, mummified fetus, musculoskeletal, neurological, pyometra, septicemia, splenomegaly, and ventral body wall abscess.

Cattle demographics were collected from individual animal health records. Arrival weights were categorized as less than 272 kg, 272–317 kg, 318–362 kg, 363–408 kg, and greater than 408 kg. Days on feed (DOFs) were categorized as 0–50 days, 51–100 days, 101–150 days, and greater than 150 days. The number of treatments prior to death were categorized as zero treatments, one treatment, two treatments, and three or more treatments.

The frequency of multiple affected SYSTs and concurrent LCATs was descriptively analyzed. Two separate models were created with the binomial outcomes being greater than one SYST or greater than one LCAT, respectively. Each case was included in each model. Using R Studio (version 2023.03.0, PBC, Boston, MA, USA), individual generalized linear mixed effect models (lme4 glmer function) were created to determine potential associations based on the probability of having greater than one SYST or LCAT with demographic factors. Fixed effects included demographic factors of arrival weight category, days on feed category, sex, and number of treatments. Univariate models were then assessed including the outcome of interest and each individual fixed effect. Both models included feedyard and year identifiers as random effects to account for the lack of independence of mortalities within the same feedyard and year.

## 3. Results

**Figure 2:**
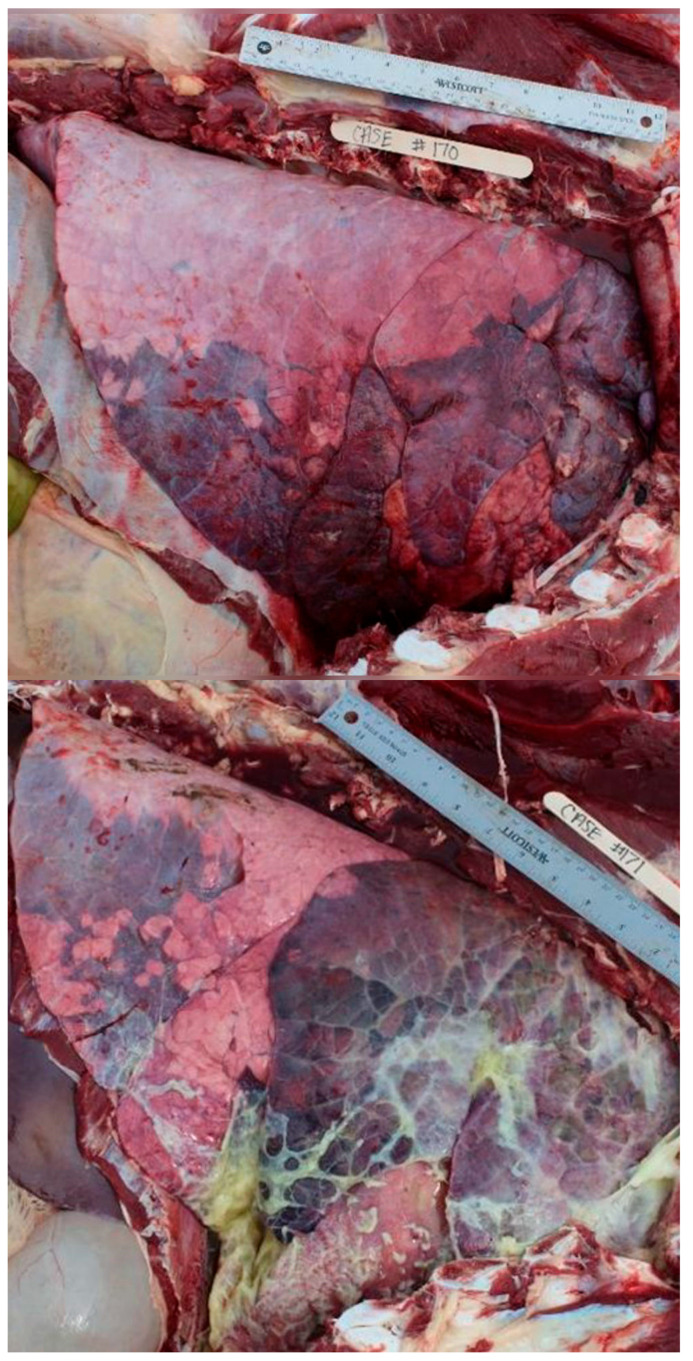
Examples of bronchopneumonia (BP) lesions. Signs of BP lesions included but were not limited to gross lung consolidation, presence of purulent materials in airways, and presence of abscesses and/or adhesions. All pictures are right-sided thoracic pictures oriented with the dorsal aspect at the top of the picture and the cranial aspect on the right.

**Figure 3:**
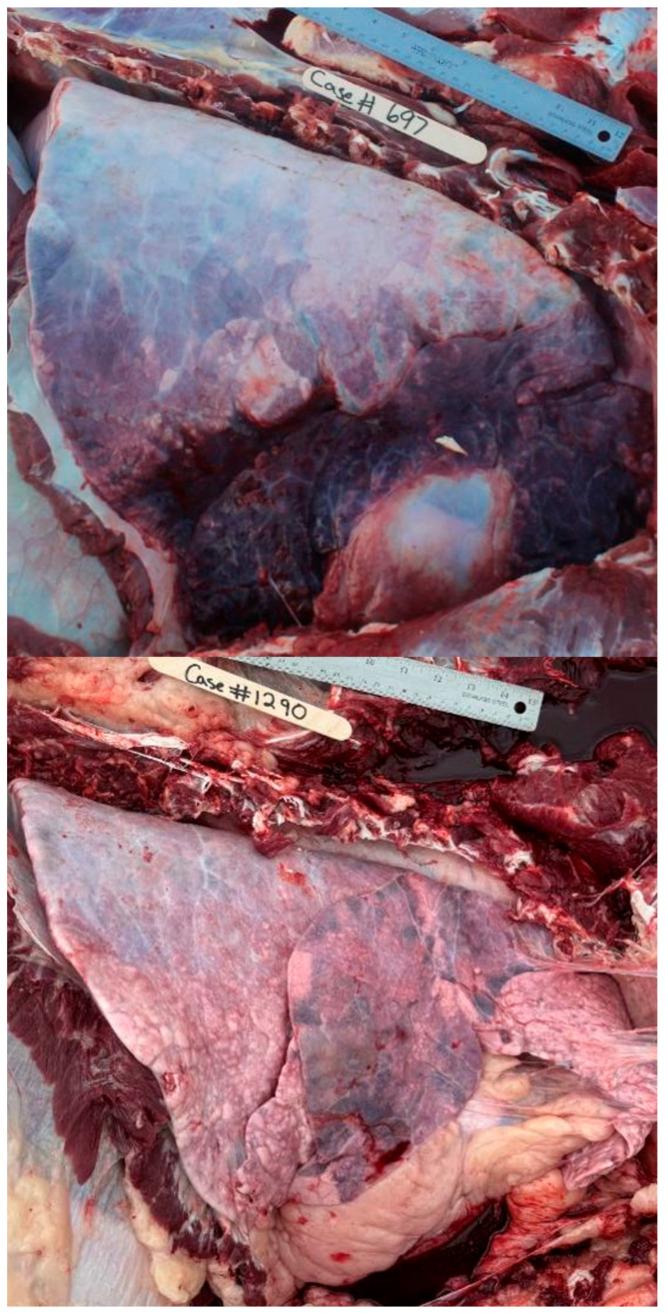
Examples of bronchopneumonia with interstitial pneumonia (BIP) lesions. Cattle with BIP lesions represented pulmonary patterns consistent with both BP and AIP in the same animal. All pictures are right-sided thoracic pictures oriented with the dorsal aspect at the top of the picture and the cranial aspect on the right.

**Figure 4:**
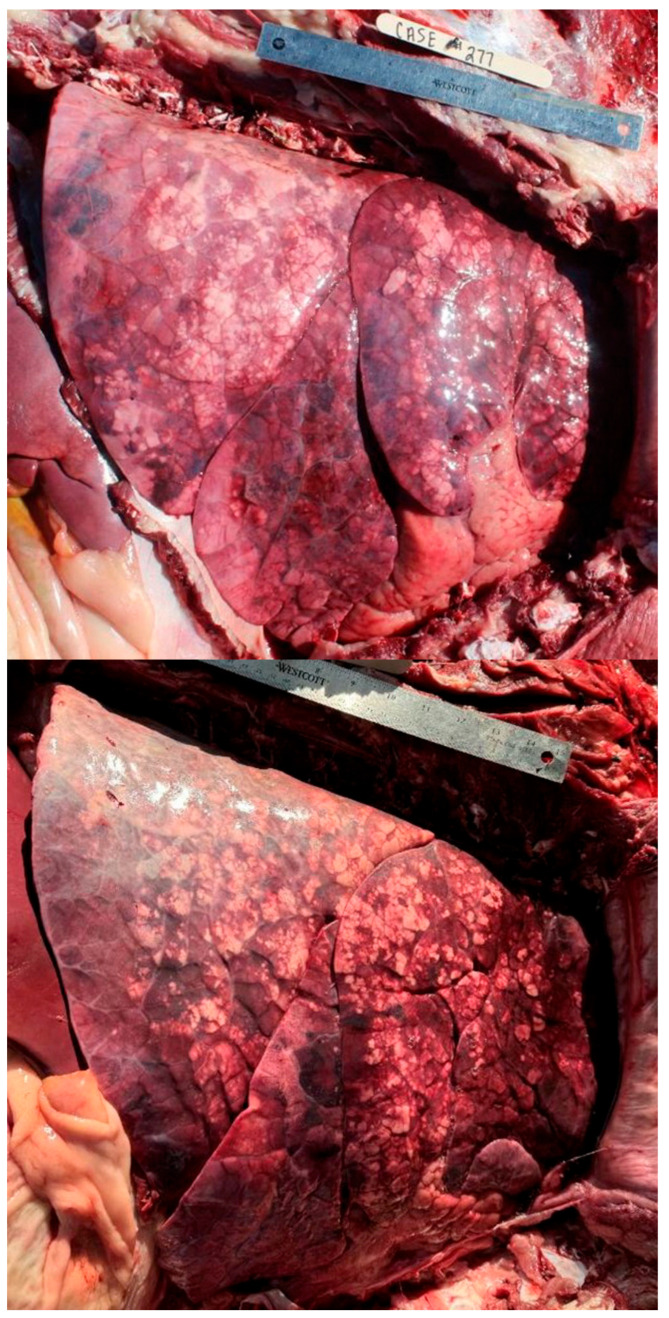
Examples of acute interstitial pneumonia (AIP) lesions. Pulmonary lesions of AIP included overinflated, wet, and heavy lungs with typical differentiation in color patterns. All pictures are right-sided thoracic pictures oriented with the dorsal aspect at the top of the picture and the cranial aspect on the right.

**Figure 5:**
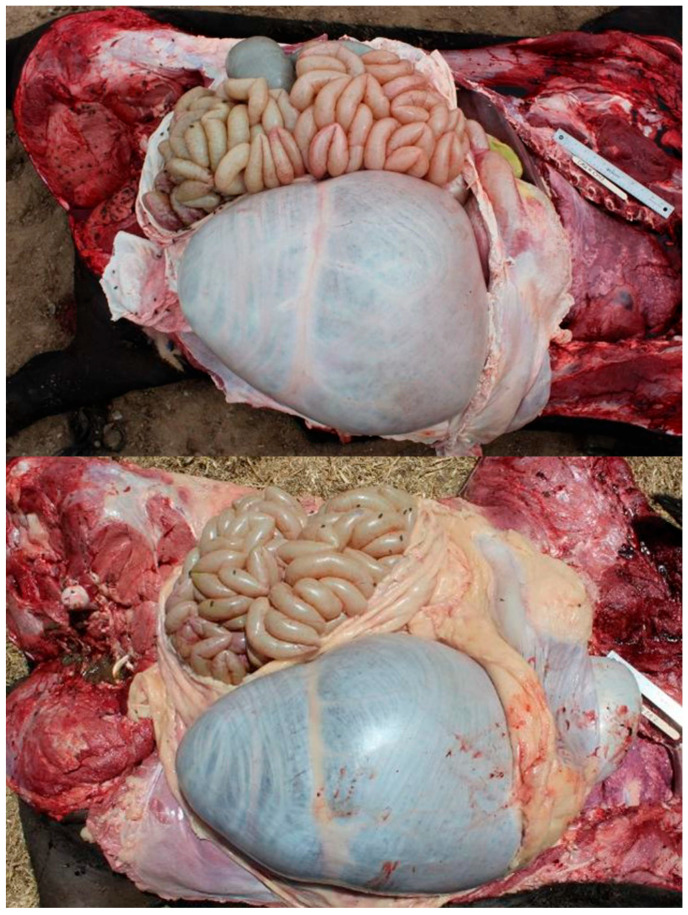
Examples of bloat lesions. Typical signs of bloat include the following: enlarged, gas-filled rumen, pale hind-quarter musculature, and compressed, pale liver and/or lungs.

**Figure 6:**
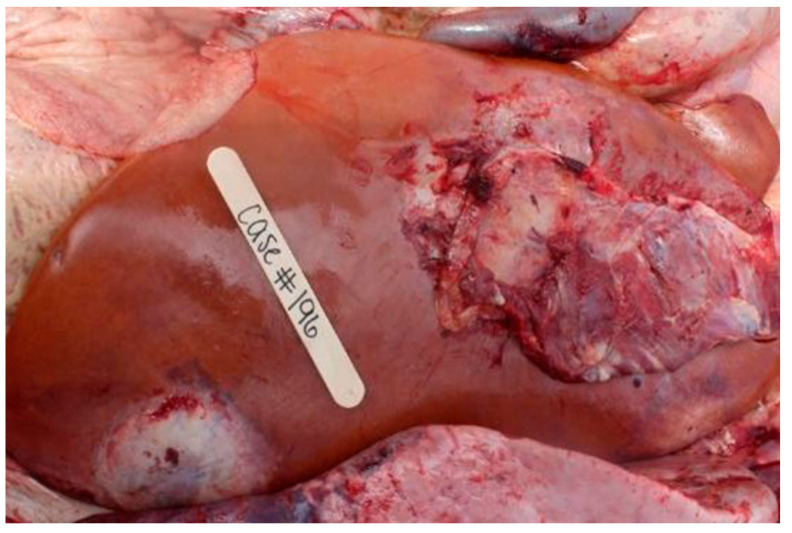

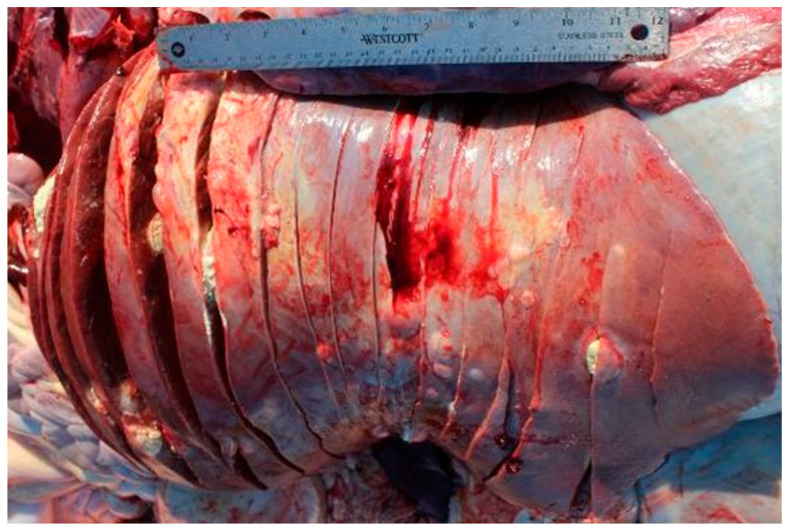
Examples of liver abscess lesions.

**Figure 7:**
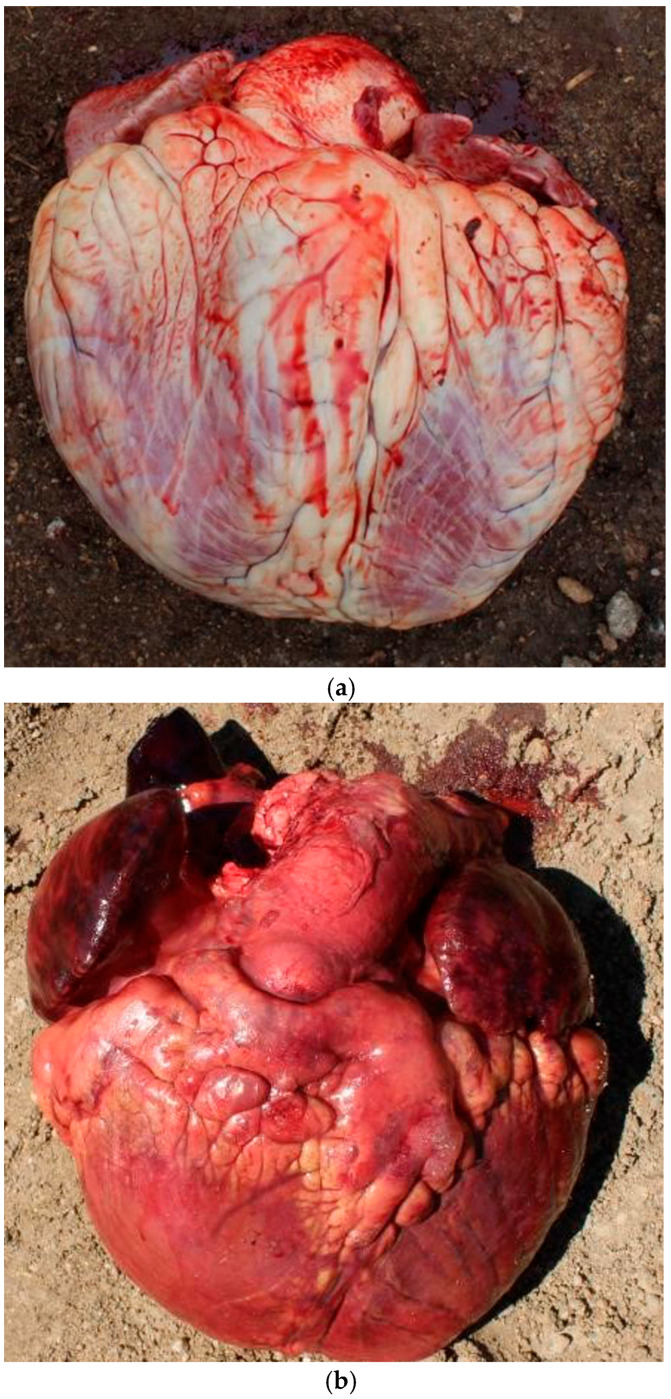

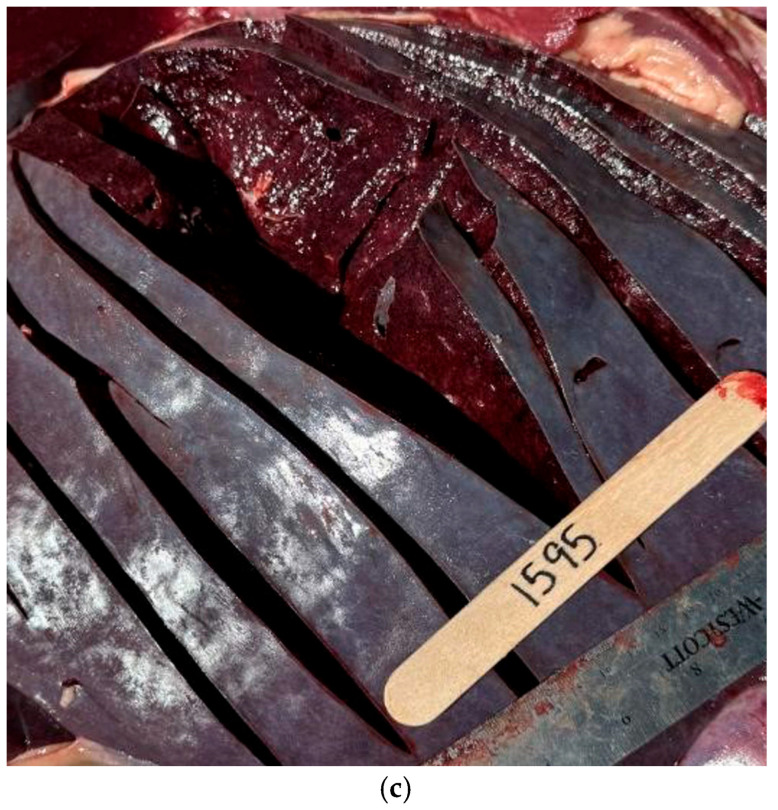
Examples of congestive heart failure (CHF) lesions. Lesions consistent with diagnosis of CHF lesions included enlarged heart (heart score 3, 4, or 5), congested (nutmeg) liver, and effusion in peritoneal, pleural, or pericardial spaces. (**a**,**b**) illustrate hearts with heart scores of 5 while (**c**) illustrates congested liver.

**Figure 8:**
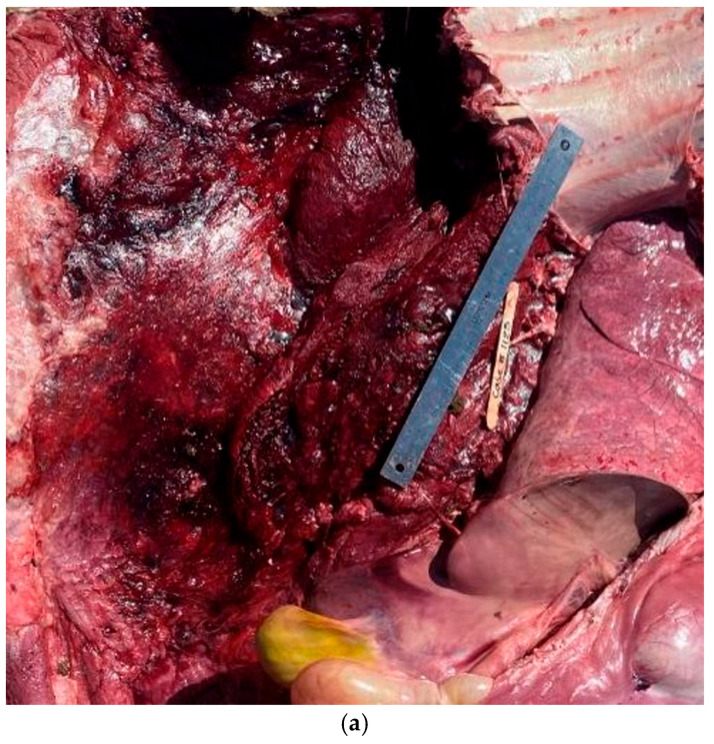

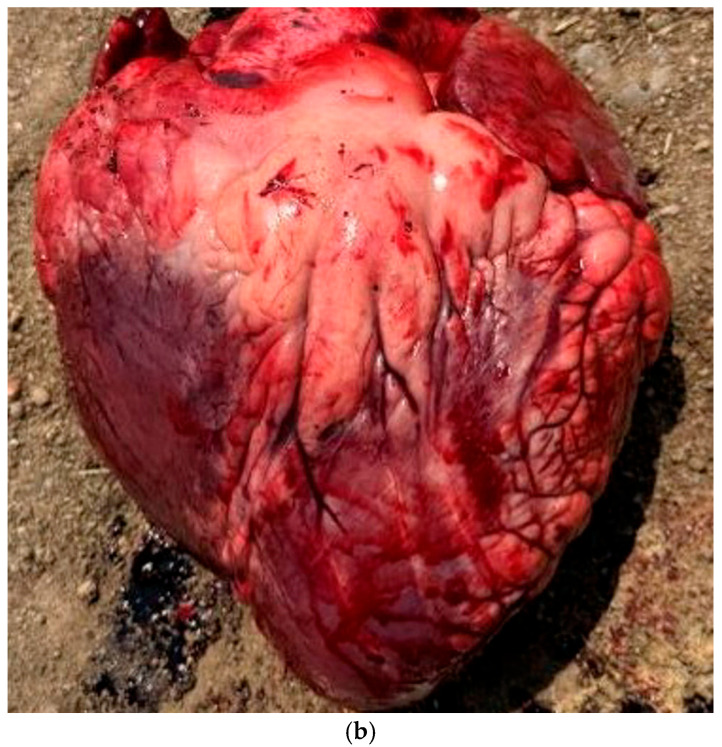
Examples of heat stress lesions. Heat stress lesions were associated with a variety of lesions including severe subcutaneous hemorrhage (**a**), cardiac petechia, and ecchymosis (**b**).

Nine hundred and thirty-one (931) necropsies were performed between the two summers of 2022 and 2023. There were forty-two cases removed due to a lack of history data or the presence of extreme autolysis, resulting in 889 cases meeting the inclusion criteria for the final analysis. The classification of cases by SYST revealed that 58% (520/889) had multiple organ systems involved. The frequency of any single lesion recorded can be seen in Table 2. Figure 2, Figure 3, Figure 4, Figure 5, Figure 6, Figure 7 and Figure 8 provide example photographs illustrating common gross pathologies associated with each of the major lesion categories. The top five coexisting SYSTs were GI and pulmonary (19%, n = 170); cardiovascular, GI, and pulmonary (6.4%, n = 57); cardiovascular and pulmonary (5.4%, n = 48); GI1, GI2, and pulmonary (5.5%, n = 49); and GI, other, and pulmonary lesions (2.6%, n = 23). The cattle demographics of these top five SYSTs are shown in Table 3. The frequency of all organ systems affected for the study population can be seen in Table S1.

Each mortality averaged 2.3 LCATs, and 72% (643/889) had more than one LCAT identified. The breakdown of individual lesion categories is shown in Table 3. The top five concurrent LCATs were BIP and other GI (8.8%, n = 72); BP and other GI (6.6%, n = 59); AIP and other GI (2.6%, n = 23); other GI and other (2.2%, n = 21); and BIP, CHF, and other GI lesions (1.8%, n = 16). The cattle demographics of these top five lesions are shown in Table 4. The frequency of all concurrent lesions in the study population can be seen in Table S2.

Univariate and multivariable analyses were performed to evaluate potential relationships between the outcomes of interest (more than one SYST or LCAT) and cattle demographic factors (arrival weight category, sex, DOFs at death, and number of previous treatments), though the significance of the fixed effects did not change between the multivariate and univariate analysis. The results presented are from the multivariate models, including all the fixed effects. The probability of a feedyard mortality having more than one SYST affected was only significantly associated (*p* < 0.05) with DOFs. Cattle that died between 0 and 50 DOFs had a lower probability of having more than one SYST affected than cattle that died between 101 and 150 DOFs (0.65 ± 0.13 SEM and 0.77 ± 0.10 SEM, respectively). Cattle in the 51–100 DOF category (0.65 ± 0.13 SEM) and >150 DOF category (0.76 ± 0.11 SEM) were not significantly different (*p* > 0.05) from any other DOF category. The probability of a feedyard mortality having greater than one LCAT was only significantly associated (*p* < 0.05) with DOF category at death. Cattle that died between 0 and 50 DOFs had a lower probability of having more than one LCAT than cattle that died between 101 and 150 DOFs (0.7 ± 0.11 SEM and 0.83 ± 0.08 SEM, respectively). Cattle in the 51–100 DOF category (0.75 ± 0.10 SEM) and >150 DOF category (0.8 ± 0.09 SEM) were not significantly different (*p* > 0.05) from any DOF category.

## 4. Discussion

Due to the negative impact that morbidities and mortalities have on feedyards, an improved understanding of the frequency of concurrent lesions could provide valuable information to decision makers for prevention and intervention. The objective of the current study was to quantify the frequency of multiple organ system involvement (pulmonary, gastrointestinal, cardiovascular, and other) and concurrent pathologic lesions in feedyard mortalities and to determine potential associations between the probability of multiple organ system involvement or concurrent lesions with demographic factors (sex, days on feed, arrival weight, and number of treatments). In the current study, we found many mortalities (72%) had more than one lesion. Some combinations of SYSTs or LCATs were more common compared to others, and there were significant associations between DOFs and having more than one SYST or LCAT.

On average, each mortality had 2.3 lesions and, grossly, most lesions were found in the pulmonary system (93% of cases) and the GI system (85% of cases). The severity of the lesions was not recorded due to the subjectiveness of grossly identifying the progress of the lesions. In the current study, most concurrent lesions were identified in separate body systems (example: pulmonary and GI) compared to concurrent lesions seen within the same body system. To the authors’ knowledge, there is no published literature focused solely on concurrently diseased body systems. Due to the nature of this cross-sectional study, there can be no inferences made on whether one lesion played a role in the development of another or if the concurrent lesions were simultaneous processes. Further research is warranted to determine possible associations or links between the involvement of multiple organ systems or concurrent lesions.

The percentage of AIP cases (9%) observed in the current study was similar to another study which also recorded the prevalence of BIP [16]. In this current study, BP was found in 39% of cases. Other studies show, of all morbidity and mortality events in feedyards, bovine respiratory disease accounts for 60–90% of cases [9,10,18]. Our study showed a smaller prevalence of BP than the other studies previously mentioned [9,10,18], but the authors presume this is due to the BIP lesion category. BIP, which has not been reported commonly as a singular disease process, includes BP and interstitial pneumonia. BIP was another common lesion found in the current study, with 32% of the cases having a BIP lesion. Another study showed that 11% of their mortalities were diagnosed as having BIP, which was the cause of death [16]. Pulmonary lesions were common in the current study population and seen often as a concurrent lesion, as they were present in four of the five top combinations of LCATs.

Concerning CHF, which is a significant cause of death in feedyard cattle in the Western Great Plains [19,20], the mortality from CHF can be as high as 7.5% in severely affected pens [21]. However, the prevalence of CHF in mortalities can differ, with a retrospective study finding that less than 1% of deaths were attributed to CHF [10]. In the current study, we found that 15% of cases had CHF lesions, but they were not necessarily severe enough to be the cause of death. The CHF lesions were commonly seen in concurrence with pulmonary lesions; however, to the authors’ knowledge, there is no published literature describing the potential association between pulmonary and cardiovascular disease. More research is warranted on the relationship between pulmonary disease and CHF.

Gastrointestinal lesions were commonly seen in the current study (85% of cases had at least one GI lesion). A study from 1994 found that digestive disorders accounted for close to 26% of deaths in beef cattle, which was less than the number of deaths due to respiratory disorders and other causes of death [22]. Another study shows that sudden deaths in feedyard cattle, often associated with digestive upsets, occur in cattle closer to market weight [23]. This also agrees with a review of disease on feedlot performance showing digestive disorders tending to occur later in the feeding period [10]. The current studies findings of a high prevalence of GI lesions, as well as a higher probability of concurrent lesions in cattle with a greater number of DOFs can suggest that there is a possible association between GI lesions and DOFs (via spending more time eating a high-starch diet).

Another finding of the current study was that the top five concurrent SYST (Table 3) and LCAT (Table 4) combinations did not represent most of the cases: only 21% and 40%, respectively. This shows that there are many different SYST and LCAT combinations, with a small number of cases in each combination group. This is likely due to differences in disease processes and severity, allowing for a variety of SYST and LCAT combinations. The most common SYST combinations included pulmonary and GI lesions. Further research is warranted on the relationship between pulmonary and GI lesions.

The length of time at the feedyard (DOFs) was the only demographic factor found to be statistically significantly associated with having more than one SYST or LCAT affected. Cattle that died within 0–50 DOFs had a lower probability of having more than one SYST or LCAT than cattle that died between 101 and 150 DOFs. One factor that could impact this result is that the longer days on feed represents a greater amount of time at risk of contracting or developing an additional disease, as well as a greater amount of time on a high-starch diet. A study from 1996 reported the relationship between mortality and DOFs, with a similar mortality rate over the entire duration of the feeding period [24]. Even though that study recorded one singular cause of death, morbidity earlier in the feeding phase could play a role in co-morbidities at necropsy later in the feeding phase. Alternatively, more common concurrent lesions in cattle with a greater number of DOFs could be associated with impacts of the inciting disease, decreasing the animal’s ability to prevent additional disease. The association between concurrent lesions and the timing of the feeding phase may also be indicative of potential challenges that have been noted in treatment response late in the feeding phase. Previous research showed that cattle treated at later than 100 DOFs had a case fatality risk of 19.1% compared to cattle treated at 31–40 or 41–50 DOFs (8.5% and 8.7%, respectively) [25]. The frequency of concurrent lesions observed later in the feeding period could decrease treatment success. For example, cattle with concurrent respiratory disease and CHF may only be treated for respiratory disease. Even if respiratory disease treatment is successful, CHF may persist. Concurrent lesions are more common in cattle with a greater number of DOFs, and further research is needed to determine the impact of concurrent lesions on treatment response.

The study’s limitations include only performing necropsies on cattle from a limited population in the Central High Plains region. This study took place during the summer months, which is associated with increased temperature and humidity. The results might differ if the study took place in the winter months or year-round. Diagnosis was determined solely by gross evaluation; thus, the classification of conditions such as necrosis may not be as accurate as they would be if histopathology was conducted. Being able to match any lesion grossly to its histopathology would solidify these findings.

Recording more than one lesion at gross necropsy could allow producers, veterinarians, and researchers to study possible associations between two or more disease processes. And, over time, this could lead to the further identification of the order of disease onset.

## 5. Conclusions

This study showed that over 70% of the cases showed more than one lesion affecting deceased feedyard cattle. GI and pulmonary lesions were the top coexisting systems identified. Realizing that the morbidity of feedyard cattle can be the result of more than one pathological disease process can better allow producers and veterinarians to create appropriate therapeutic interventions and health protocols. Even though the cause of death might be predominantly attributed to one organ system or lesion, recording all lesions at necropsy could be beneficial in the consideration of all disease processes and possible interactions. In the future, more refined diagnostic tools at necropsy could improve health protocols for morbidities.

## Figures and Tables

**Table 1:** Necropsy evaluation variables outside the normal limits that were observed and recorded during 889 gross necropsies at Central High Plain of feedyard mortalities in the summers of 2022 and 2023.

Area of Examination:		Options, if Not Within Normal Limits:
External:	Sex	Steer, Heifer
	Breed	Native, Holstein, Mexican, Beef × Dairy
	Body condition	Thin, Moderate, Fat
	Lactation status	Lactating, Not Lactating
	Mastitis status	Mastitis, No Mastitis
Integumentary:	Oral cavity	Ulcers, Blunted Papillae, Petechia
Esophagus, Trachea, Heart:	Esophagus	Ulcers, Hemorrhage
	Larynx/Pharynx	Necrosis, Infection, Ulcers, Hemorrhage, Fibrinous
	Tracheal contents/Mucosa	Froth, Reflux, Hemorrhage, Fibrinonecrotic, Ulcers, Edema, Infection
	Pericardial effusion/Sac	Fibrinous, Mucoid, Purulent, Sanguineous, Serous, Serosanguineous, Thickened, Adhesions
	Heart score	1, 2, 3, 4, 5
	Heart circumference (cm)	
	Heart weight (g)	
	Myocardium/Endocardium	Necrosis, Petechia, Ecchymosis, Dilated Ventricle, Myocarditis, Endocarditis
Lungs:	Pleural effusion	Fibrinous, Mucoid, Purulent, Sanguineous, Serous, Serosanguineous
	Pleural effusion volume	Mild, Moderate, Severe
	Pulmonary pathology	Bronchopneumonia, Fibrinous, Pleuritis, Acute Interstitial Pneumonia, Bronchopneumonia with Interstitial Pneumonia, Granulomatous, Embolic Pneumonia, Pulmonary Hemorrhage/Petechia, Pulmonary Edema
	Abscesses	Small, Medium, Large/Few, Moderate, Many
	Adhesions	Present, Absent
GI Tract:	Small intestine (SI) serosa	Red, Black
	SI mucosa	Ulcer, Hemorrhage, Thickened
	SI content	Gas, Fluid
	SI lesion	Obstructed, Inflamed, Parasites, Neoplasia
	Large intestine (LI) serosa	Red, Black
	LI mucosa	Ulcer, Hemorrhage, Thickened
	LI content	Gas, Fluid
	LI lesion	Obstructed, Inflamed, Parasites, Neoplasia
	Rumen score	1, 2, 3, 4, 5
	Rumen contents	Froth, Full, Empty, Bloated, Fluid
	Rumen mucosa	Ulcer, Hemorrhage, Thickened, Parakeratosis
	Abomasal mucosa	Ulcer, Hemorrhage, Thickened
Abdomen:	Mesenteric lymph nodes	Enlarged, Hemorrhage
	Peritoneal fluid	Fibrinous, Mucoid, Purulent, Sanguineous, Serous, Serosanguineous
	Adhesions	Liver, SI, LI, Forestomach, Other
	Liver	Nutmeg, Congested, Jaundiced, Pale, Scars, Flukes, Enlarged
	Liver abscess grade	O, A, A+
	Kidney	Enlarged, Contracted, Infarcts, Petechia, Abscess, Infection
	Bladder/urine	Ecchymosis, Calculi, Cystitis, Pale, Dark Brown, Hemorrhage, Exudate, Ruptured, Stones
	Reproductive	Infected, Adhesions, Pregnant
	Spleen	Swollen, Contracted, Infarcted, Enlarged
Musculoskeletal:	Lesion	Swollen, Trauma, Muscle Necrosis, Ecchymosis
Final Diagnosis:		

**Table 2:** Frequency of organ systems affected (SYST) by lesion category (LCAT) from 889 Central High Plain feedyard mortalities at gross necropsy in the summers of 2022 and 2023. Abbreviations in the figure include AIP (acute interstitial pneumonia), BP (bronchopneumonia), BIP (bronchopneumonia with an interstitial pattern), GI (gastrointestinal), and CHF (congestive heart failure).

SYST:	LCAT:	Frequency of Lesion (n)	Percent of Cases (n/889 × 100)
Pulmonary	BP	354	38.8%
	BIP	281	31.6%
	AIP	76	8.6%
	Other Pulmonary	122	13.7%
	Total Pulmonary	824	92.7%
GI	Bloat	83	9.3%
	Liver Abscess	47	5.3%
	Other GI	625	70.3%
	Total GI	755	84.9%
Cardiovascular	CHF	136	15.3%
	Heat Stress	40	4.5%
	Other Cardiovascular	141	15.9%
	Total Cardiovascular	317	35.7%
Other	Other	144	16.2%
	Total other	144	16.2%
Total lesions		2040	230%

**Table 3:** Top 5 concurrent organ systems (SYST) affected and cattle demographics from 889 Central High Plain feedyard mortalities at gross necropsy in the summers of 2022 and 2023. In this table, GI represents gastrointestinal lesions, GI1 represents the first gastrointestinal lesion, and GI2 represents the second gastrointestinal lesion.

	Top 5 Concurrent Systems (SYST)				
	GI and Pulmonary	Cardiovascular, GI, and Pulmonary	Cardiovascular and Pulmonary	GI1, GI2, and Pulmonary	GI, Other, and Pulmonary
	(n = 170)	(n = 57)	(n = 48)	(n = 49)	(n = 23)
Sex (%)					
Steer	31%	28%	33%	29%	26%
Heifer	69%	72%	67%	71%	74%
Arrival Weight (kg; avg)	324.6	331.4	324.4	323.1	353.8
Days on Feed (avg)	90	113	97	101	57
Number of Treatments (avg)	2	1	2	1	2

**Table 4:** Top 5 concurrent lesion categories (LCATs) and cattle demographics from 889 Central High Plain feedyard mortalities at gross necropsy in the summers of 2022 and 2023.

	Top 5 Concurrent Lesion Categories (LCATs)				
	BIP and Other GI	BP and Other GI	AIP and Other GI	Other GI and Other	BIP, CHF, and Other GI
	(n = 72)	(n = 59)	(n = 23)	(n = 21)	(n = 16)
Sex (%)					
Steer	25%	31%	35%	40%	13%
Heifer	75%	69%	65%	60%	87%
Arrival Weight (kg; avg)	326.2	324.5	321.3	329.5	325.3
Days on Feed (avg)	94	70	106	72	120
Number of Treatments (avg)	2	2	2	2	1

## Data Availability

The data used in this research were provided by cooperating entities and are not publicly available due to confidentiality and anonymity agreements.

## Supplementary Materials

The following supporting information can be downloaded at: https://www.mdpi.com/article/10.3390/vetsci12070666/s1, Table S1: Frequency of total concurrent organ systems affected; Table S2: Frequency of total concurrent lesions.

## Abbreviations

The following abbreviations are used in this manuscript:

DOFs	Days on Feed
BIP	Bronchopneumonia with Interstitial Pneumonia
AIP	Acute Interstitial Pneumonia
BP	Bronchopneumonia
GI	Gastrointestinal
CHF	Congestive Heart Failure
SYST	Organ System
LCAT	Lesion Category
