# Supplementary material for: Determining Frequency of Multiple Organ System Involvement and Concurrent Lesions Identified in Feedyard Mortalities and Potential Associations with Cattle Demographics
Source: Vet Sci. 2025 Jul 15;12(7):666. doi: 10.3390/vetsci12070666 (PMC12300491; doi:10.3390/vetsci12070666)
Supplement: Supplementary file 1 [file vetsci-12-00666-s001.zip › concurrent table S1.pdf]

Table S1: Frequency of total concurrent organ systems affected

**Concurrent Organ Systems (SYST)**	**Count of cases (n)**
GI, Pulmonary	170
Cardiovascular, GI, Pulmonary	57
Cardiovascular, Pulmonary	48
GI, GI, Pulmonary	49
GI, Other, Pulmonary	23
GI, Other	20
GI, Pulmonary, Pulmonary	18
Cardiovascular, GI, GI, Pulmonary	17
Cardiovascular, GI	15
Other, Pulmonary	14
GI, GI	13
GI, GI, GI, Pulmonary	12
Cardiovascular, Cardiovascular, GI, Pulmonary	9
Cardiovascular, GI, Pulmonary, Pulmonary	9
GI, GI, Other, Pulmonary	7
Cardiovascular, GI, GI, Pulmonary, Pulmonary	6
Cardiovascular, Cardiovascular, GI	5
Cardiovascular, Cardiovascular, GI, GI, Pulmonary	5
Cardiovascular, Cardiovascular, Pulmonary	5
Cardiovascular, GI, GI, GI, Pulmonary	5
GI, GI, Pulmonary, Pulmonary	5
Pulmonary, Pulmonary	5
Cardiovascular, Cardiovascular, GI, Pulmonary, Pulmonary	4
Other, Other, Pulmonary	4
Cardiovascular, Cardiovascular, Pulmonary, Pulmonary	3
Cardiovascular, GI, GI, Other, Pulmonary	3

Cardiovascular, GI, Other	3
Cardiovascular, Pulmonary, Pulmonary	3
GI, GI, Other	3
Other, Pulmonary, Pulmonary	3
Cardiovascular, Cardiovascular, Cardiovascular, Pulmonary	2
Cardiovascular, Cardiovascular, GI, Other, Pulmonary	2
GI, Other, Other	2
GI, Other, Other, Pulmonary	2
GI, Other, Pulmonary, Pulmonary	2
Cardiovascular, Cardiovascular, Cardiovascular, GI, Pulmonary	1
Cardiovascular, Cardiovascular, Cardiovascular, GI, Pulmonary, Pulmonary	1
Cardiovascular, Cardiovascular, Cardiovascular, Pulmonary, Pulmonary	1
Cardiovascular, Cardiovascular, GI, GI, GI, GI, Other, Pulmonary	1
Cardiovascular, Cardiovascular, GI, GI, GI, Pulmonary, Pulmonary	1
Cardiovascular, Cardiovascular, GI, Other, Pulmonary, Pulmonary	1
Cardiovascular, Cardiovascular, GI, Pulmonary, Pulmonary, Pulmonary	1
Cardiovascular, Cardiovascular, Other, Pulmonary	1
Cardiovascular, GI, GI	1
Cardiovascular, GI, GI, GI, GI, Pulmonary, Pulmonary	1
Cardiovascular, GI, GI, Other	1
Cardiovascular, GI, GI, Other, Pulmonary, Pulmonary	1
Cardiovascular, GI, Other, Other, Pulmonary, Pulmonary	1
Cardiovascular, GI, Other, Pulmonary	1
Cardiovascular, GI, Other, Pulmonary, Pulmonary, Pulmonary	1
Cardiovascular, GI, Pulmonary, Pulmonary, Pulmonary	1
Cardiovascular, Other	1

Cardiovascular, Other, Other, Pulmonary	1
Cardiovascular, Other, Pulmonary	1
Cardiovascular, Other, Pulmonary, Pulmonary	1
Cardiovascular, Pulmonary, GI	1
GI, Cardiovascular	1
GI, GI, GI	1
GI, GI, GI, GI, Pulmonary	1
GI, GI, GI, Other, Pulmonary	1
GI, GI, GI, Other, Pulmonary, Pulmonary	1
GI, GI, GI, Pulmonary, Pulmonary	1
GI, GI, Other, Other, Pulmonary	1
GI, Other, Other, Pulmonary, Pulmonary	1

Frequency of SYST from 889 central high plain feedyard mortalities at gross necropsy in the summers of 2022 and 2023, in descending order from most common to least common. An individual organ system can be counted multiple times to account for different lesions within the organ system.
