# Supplementary material for: Determining Frequency of Multiple Organ System Involvement and Concurrent Lesions Identified in Feedyard Mortalities and Potential Associations with Cattle Demographics
Source: Vet Sci. 2025 Jul 15;12(7):666. doi: 10.3390/vetsci12070666 (PMC12300491; doi:10.3390/vetsci12070666)
Supplement: Supplementary file 1 [file vetsci-12-00666-s001.zip › concurrent table S2.pdf]

Table S2: Frequency of total concurrent lesions

Concurrent Lesion Categories (LCAT)	Count of cases (n)
BIP, GI	72
BP, GI	59
AIP, GI	23
GI, Other	21
BIP, CHF, GI	16
BIP, GI, GI	14
BIP, Other Cardiovascular	13
Bloat, GI	13
BP, GI, Other	13
BIP, CHF	12
Bloat, BP, GI	11
BP, GI, GI	11
BP, Other	11
Bloat, BP	10
BIP, GI, Other Pulmonary	9
BP, GI, Other Cardiovascular	9
BP, GI, Other Pulmonary	8
BP, Other Cardiovascular	8
GI, GI	8
BP, CHF, GI	7
AIP, Other Cardiovascular	6
BIP, GI, Other Cardiovascular	6
BIP, Other	6
AIP, CHF, GI	5
BP, CHF	5

BP, Liver Abscess	5
BP, Other Pulmonary	5
GI, Other, Other Pulmonary	5
GI, Other Cardiovascular	5
AIP, GI, Other Cardiovascular	4
BIP, GI, GI, GI	4
Bloat, CHF	4
Bloat, GI, GI	4
BP, CHF, GI, GI	4
BP, CHF, GI, GI, Other Cardiovascular	4
BP, CHF, GI, Other Pulmonary	4
BP, Heat Stress	4
CHF, GI	4
GI, GI, GI	4
Other, Other Pulmonary	4
AIP, CHF	3
AIP, GI, GI	3
BIP, CHF, GI, GI, Other Pulmonary	3
BIP, GI, Liver Abscess	3
BIP, GI, Other	3
BIP, Liver Abscess	3
Bloat, Other Cardiovascular	3
BP, CHF, Other Cardiovascular, Other Pulmonary	3
BP, GI, GI, Other	3
BP, GI, GI, Other Pulmonary	3
BP, Other, Other	3
BP, Other, Other Pulmonary	3
CHF, GI, Heat Stress	3

GI, Heat Stress	3
Heat Stress, Liver Abscess	3
AIP, CHF, GI, GI	2
AIP, CHF, GI, Other Cardiovascular	2
BIP, Bloat, GI	2
BIP, CHF, GI, GI	2
BIP, CHF, GI, GI, GI	2
BIP, CHF, GI, Other Cardiovascular	2
BIP, CHF, GI, Other Cardiovascular, Other Pulmonary	2
BIP, CHF, GI, Other, Other Cardiovascular	2
BIP, CHF, Liver Abscess	2
BIP, GI, GI, Other Cardiovascular	2
BIP, GI, GI, Other Pulmonary	2
BIP, GI, Liver Abscess, Other Cardiovascular	2
Bloat, BP, CHF, GI, Other	2
Bloat, BP, GI, GI	2
BP, GI, GI, GI	2
BP, GI, Heat Stress	2
BP, GI, Liver Abscess	2
BP, GI, Liver Abscess, Other Cardiovascular	2
BP, GI, Other Cardiovascular, Other Pulmonary	2
CHF, GI, Other Pulmonary	2
GI, GI, Other Cardiovascular	2
GI, GI, Other, Other Pulmonary	2
GI, Other Cardiovascular, Other Cardiovascular	2
GI, Other Cardiovascular, Other Pulmonary	2
GI, Other Pulmonary	2
GI, Other, Other Cardiovascular	2

Other, Other Cardiovascular, Other Pulmonary	2
AIP, Bloat	1
AIP, Bloat, CHF, GI, GI	1
AIP, CHF, Heat Stress	1
AIP, CHF, Other Cardiovascular	1
AIP, CHF, Other Cardiovascular, Other Cardiovascular, Other Pulmonary	1
AIP, GI, GI, Heat Stress, Other Pulmonary	1
AIP, GI, GI, Liver Abscess	1
AIP, GI, GI, Other Cardiovascular, Other Pulmonary	1
AIP, GI, Heat Stress, Other Pulmonary	1
AIP, GI, Liver Abscess	1
AIP, GI, Other	1
AIP, GI, Other Cardiovascular, Other Pulmonary	1
AIP, Other Pulmonary	1
BIP, CHF, GI, GI, GI, GI, Other, Other Cardiovascular	1
BIP, CHF, GI, GI, Heat Stress	1
BIP, CHF, GI, Liver Abscess	1
BIP, CHF, GI, Liver Abscess, Other Pulmonary	1
BIP, CHF, GI, Other	1
BIP, CHF, GI, Other Pulmonary, Other Pulmonary	1
BIP, CHF, Other Cardiovascular	1
BIP, CHF, Other Cardiovascular, Other Cardiovascular	1
BIP, CHF, Other Pulmonary	1
BIP, GI, GI, GI, GI	1
BIP, GI, GI, GI, Other, Other Pulmonary	1
BIP, GI, GI, Heat Stress	1
BIP, GI, GI, Liver Abscess	1
BIP, GI, GI, Liver Abscess, Other	1

BIP, GI, GI, Other	1
BIP, GI, GI, Other Cardiovascular, Other Cardiovascular	1
BIP, GI, Heat Stress	1
BIP, GI, Other Cardiovascular, Other Cardiovascular	1
BIP, GI, Other, Other Pulmonary	1
BIP, Liver Abscess, Other, Other Cardiovascular, Other Pulmonary, Other Pulmonary	1
BIP, Other Pulmonary	1
BIP, Other, GI	1
Bloat, BP, CHF, GI, GI, Other Cardiovascular, Other Pulmonary	1
Bloat, BP, GI, Liver Abscess	1
Bloat, BP, Other	1
Bloat, BP, Other Cardiovascular	1
Bloat, CHF, Heat Stress	1
Bloat, CHF, Other	1
Bloat, GI, GI, Other Pulmonary	1
Bloat, GI, Liver Abscess	1
Bloat , GI, Other	1
Bloat, Liver Abscess	1
Bloat, Liver Abscess, Other Cardiovascular	1
Bloat, Other Pulmonary	1
BP, Bloat, GI, GI	1
BP, CHF, GI, GI, GI	1
BP, CHF, GI, GI, Other, Other Pulmonary	1
BP, CHF, GI, Heat Stress	1
BP, CHF, GI, Other Cardiovascular, Other Pulmonary	1
BP, CHF, GI, Other Cardiovascular, Other Pulmonary, Other Pulmonary	1
BP, CHF, Liver Abscess, Other Cardiovascular	1
BP, CHF, Liver Abscess, Other Pulmonary	1

BP, CHF, Other Cardiovascular	1
BP, CHF, Other Pulmonary	1
BP, CHF, Other, Other	1
BP, CHF, Other, Other Pulmonary	1
BP, GI, GI, GI, GI, Other Cardiovascular, Other Pulmonary	1
BP, GI, GI, GI, Other Pulmonary	1
BP, GI, GI, Heat Stress	1
BP, GI, GI, Heat Stress, Other	1
BP, GI, GI, Other, Other Pulmonary	1
BP, GI, Heat Stress, Other Cardiovascular, Other Pulmonary	1
BP, GI, Other Cardiovascular, Other Cardiovascular	1
BP, GI, Other, Other	1
BP, GI, Other, Other Cardiovascular	1
BP, GI, Other, Other Cardiovascular, Other Cardiovascular, Other Pulmonary	1
BP, Heat Stress, Other	1
BP, Heat Stress, Other Pulmonary	1
BP, Liver Abscess, Other	1
BP, Liver Abscess, Other, Other, Other Pulmonary	1
BP, Other Cardiovascular, GI	1
BP, Other, Other, Other Cardiovascular	1
CHF, GI, GI, Liver Abscess, Other Pulmonary	1
CHF, GI, Other Cardiovascular, Other Cardiovascular, Other Pulmonary	1
CHF, GI, Other, Other, Other Pulmonary, Other Pulmonary	1
CHF, Liver Abscess, Other Cardiovascular, Other Cardiovascular, Other Pulmonary, Other Pulmonary	1
CHF, Liver Abscess, Other Cardiovascular, Other Pulmonary	1
CHF, Liver Abscess, Other Pulmonary	1
CHF, Other	1

CHF, Other Cardiovascular, Other Cardiovascular, Other Pulmonary	1
CHF, Other Cardiovascular, Other Pulmonary	1
CHF, Other Pulmonary	1
GI, GI, GI, Liver Abscess, Other Pulmonary	1
GI, GI, Heat Stress, Other	1
GI, GI, Heat Stress, Other Pulmonary	1
GI, GI, Other	1
GI, GI, Other Pulmonary	1
GI, GI, Other, Other	1
GI, GI, Other, Other, Other Pulmonary	1
GI, Heat Stress, Other Cardiovascular	1
GI, Liver Abscess, Other	1
GI, Liver Abscess, Other, Other Cardiovascular	1
GI, Liver Abscess, Other, Other Pulmonary	1
GI, Other, Other, Other Pulmonary	1
Heat Stress, Other Cardiovascular	1
Heat Stress, Other Pulmonary	1
Liver Abscess, Other, Other	1
Liver Abscess, Other, Other Cardiovascular	1
Other Cardiovascular, Other Pulmonary	1
Other Pulmonary, Other Pulmonary, GI	1
Other, Other Cardiovascular, Other Cardiovascular, Other Pulmonary	1

Frequency of LCAT from 889 central high plain feedyard mortalities at gross necropsy in the summers of 2022 and 2023, in descending order from most common to least common.
